# Diagnostic value of amyloid-PET and tau-PET: a head-to-head comparison

**DOI:** 10.1007/s00259-021-05246-x

**Published:** 2021-02-27

**Authors:** Daniele Altomare, Camilla Caprioglio, Frédéric Assal, Gilles Allali, Aline Mendes, Federica Ribaldi, Kelly Ceyzeriat, Marta Martins, Szymon Tomczyk, Sara Stampacchia, Alessandra Dodich, Marina Boccardi, Christian Chicherio, Giovanni B. Frisoni, Valentina Garibotto

**Affiliations:** 1 Laboratory of Neuroimaging of Aging (LANVIE), University of Geneva, Geneva, Switzerland; 2 Memory Clinic, Geneva University Hospitals, Geneva, Switzerland; 3 Division of Neurology, Department of Clinical Neurosciences, Geneva University Hospitals, Geneva, Switzerland; 4 Faculty of Medicine, University of Geneva, Geneva, Switzerland; 5 Department of Neurology, Division of Cognitive & Motor Aging, Albert Einstein College of Medicine, Yeshiva University, Bronx, NY USA; 6 Division of Geriatrics, Department of Rehabilitation and Geriatrics, Geneva University Hospitals, Geneva, Switzerland; 7 Laboratory of Alzheimer’s Neuroimaging and Epidemiology (LANE), Saint John of God Clinical Research Centre, Brescia, Italy; 8 Department of Molecular and Translational Medicine, University of Brescia, Brescia, Italy; 9 Laboratory of Neuroimaging and Innovative Molecular Tracers (NIMTlab), Geneva University Neurocenter and Faculty of Medicine, University of Geneva, Geneva, Switzerland; 10 Division of Adult Psychiatry, Department of Psychiatry, Geneva University Hospitals, Geneva, Switzerland; 11 Center for Mind/Brain Sciences (CIMeC), University of Trento, Rovereto, Italy; 12 Late Translational Dementia Research Group, German Center for Neurodegenerative Diseases (DZNE), Rostock-Greifswald site, Rostock, Germany; 13 Division of Nuclear Medicine and Molecular Imaging, Geneva University Hospitals, Geneva, Switzerland

**Keywords:** Amyloid, Tau, PET, Florbetapir, Flutemetamol, Flortaucipir

## Abstract

**Purpose:**

Assess the individual and combined diagnostic value of amyloid-PET and tau-PET in a memory clinic population.

**Methods:**

Clinical reports of 136 patients were randomly assigned to two diagnostic pathways: AMY-TAU, amyloid-PET is presented before tau-PET; and TAU-AMY, tau-PET is presented before amyloid-PET. Two neurologists independently assessed all reports with a balanced randomized design, and expressed etiological diagnosis and diagnostic confidence (50–100%) three times: (i) at baseline based on the routine diagnostic workup, (ii) after the first exam (amyloid-PET for the AMY-TAU pathway, and tau-PET for the TAU-AMY pathway), and (iii) after the remaining exam. The main outcomes were changes in diagnosis (from AD to non-AD or vice versa) and in diagnostic confidence.

**Results:**

Amyloid-PET and tau-PET, when presented as the first exam, resulted in a change of etiological diagnosis in 28% (*p* = 0.006) and 28% (*p* < 0.001) of cases, and diagnostic confidence increased by 18% (*p* < 0.001) and 19% (*p* < 0.001) respectively, with no differences between exams (*p* > 0.05). We observed a stronger impact of a negative amyloid-PET versus a negative tau-PET (*p* = 0.014). When added as the second exam, amyloid-PET and tau-PET resulted in a further change in etiological diagnosis in 6% (*p* = 0.077) and 9% (*p* = 0.149) of cases, and diagnostic confidence increased by 4% (*p* < 0.001) and 5% (*p* < 0.001) respectively, with no differences between exams (*p* > 0.05).

**Conclusion:**

Amyloid-PET and tau-PET significantly impacted diagnosis and diagnostic confidence in a similar way, although a negative amyloid-PET has a stronger impact on diagnosis than a negative tau-PET. Adding either of the two as second exam further improved diagnostic confidence.

**Trial number:**

PB 2016-01346.

**Supplementary Information:**

The online version contains supplementary material available at 10.1007/s00259-021-05246-x.

## Introduction

The pathological accumulation of aggregated proteins in the brain represents a hallmark of most neurodegenerative diseases. Specifically, amyloid and tau are the two main proteins involved in Alzheimer’s disease (AD). AD pathophysiological changes precede clinical symptoms by about 20 years [1]. Therefore, the use of amyloid and tau biomarkers allows to identify AD in its early phases, i.e., in patients with mild cognitive impairment (MCI), and make differential diagnosis in cases with atypical presentations [1].

Thus, the recent development of PET tracers allowing to assess and quantify brain amyloid and tau deposition in vivo represented a breakthrough for research in this field. Indeed, such pivotal tools have been recently incorporated in research frameworks defining AD based only on the abnormal deposition of both proteins irrespective of the clinical profile [2, 3].

The first study on humans involving an amyloid-PET ligand was published 17 years ago [4], and converging evidence suggests that amyloid-PET is a helpful support for clinicians, leading to changes of diagnoses and treatment plan based on traditional work-up and increase of confidence on etiological diagnosis [5]. Tau-PET was more recently introduced [6], and 18F-Flortaucipir, the most widely used first-generation tau-PET tracer, has shown partial analytical validity and preliminary evidence of clinical validity [7] and has recently been approved by the FDA for estimating density and distribution of tau neurofibrillary tangles in vivo [8], but evidence on the diagnostic value of tau-PET is not available yet.

To date, evidence on the relative and combined diagnostic value of biomarkers assessing amyloid and tau is scanty. Recently, Ramusino and colleagues compared the relative incremental diagnostic value of amyloid-PET and CSF (Aβ_42_, p-tau, t-tau), showing that amyloid-PET induces greater changes in the diagnosis of AD patients and an overall increase of diagnostic confidence when performed both as the first investigation and even after CSF, possibly because of the higher negative predictive value of amyloid-PET [9]. However, no studies have assessed the diagnostic value of amyloid-PET and tau-PET (and their combination) in clinical practice.

This study aims to provide preliminary evidence on the diagnostic value, in terms of changes of etiological diagnosis and diagnostic confidence, of amyloid-PET and tau-PET performed using 18F-Flortaucipir, both individually and in combination, in an unselected memory clinic population. In order to investigate the impact of amyloid-PET and tau-PET in patients with different cognitive stages, the analyses were replicated in subsamples including only patients with subjective cognitive decline (SCD), or only those with MCI, or only those with dementia. Our a priori hypothesis is that tau-PET has a greater diagnostic value than amyloid-PET.

## Material and methods

### Participants

The participants were 136 patients with cognitive complaints recruited consecutively and evaluated at the Geneva Memory Clinic, who underwent, from November 2015 to July 2020, a diagnostic workup including clinical and neuropsychological assessments, MRI, and amyloid-PET and tau-PET within an ongoing prospective research study. The study focused on the interplay between amyloidosis and tau-related neurodegeneration was approved by the local ethic committee (PB 2016–01346) and has been conducted in accordance with the principles of the Declaration of Helsinki and the International Conference on Harmonization Good Clinical Practice. Each subject provided a voluntary written informed consent for the participation in the study. The mean time interval between amyloid-PET and tau-PET is 5 months (SD = 5 months).

### Study design

**Fig. 1:**
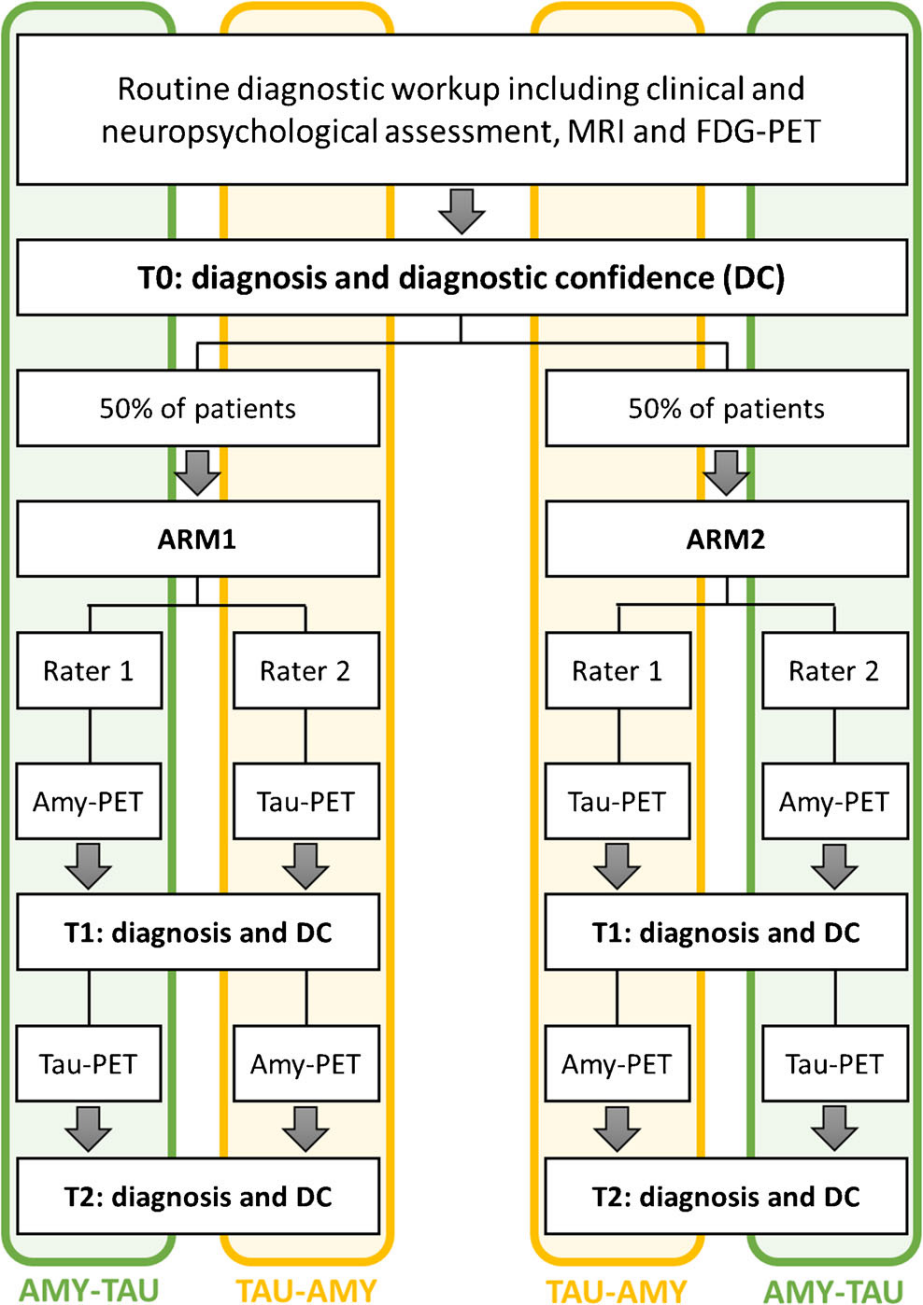
Study design

Figure 1 illustrates the study design. To assess the relative incremental diagnostic value of amyloid-PET and tau-PET, the data of the study participants were retrospectively randomized into two study arms resulting in two distinct diagnostic pathways: (i) AMY-TAU, amyloid-PET followed by tau-PET; or (ii) TAU-AMY, tau-PET followed by amyloid-PET. Two independent raters (FA and GA, both neurologists with ≥15 years of experience in the field of neurodegenerative disorders) blindly assessed patients’ clinical reports three times with a balanced randomization of patients relative to the arm and the order of the two PET exams (Fig. 1). At T0 (baseline), the two raters received the anonymized clinical reports including clinical and neuropsychological assessments as well as the MRI report, and were asked to indicate a cognitive stage (e.g., subjective cognitive decline (SCD) [10], MCI [11], dementia [12]) and an etiological diagnosis and rate their diagnostic confidence (from 50%, max uncertainty, to 100%, max certainty) in such etiological diagnosis for each patient. At T1, the two raters were asked to revise the baseline etiological diagnosis and diagnostic confidence for each patient based on the first exam received (either amyloid-PET or tau-PET, according to the study design): for patients in ARM1, the first rater received the amyloid-PET report as the first exam, while the second rater received the tau-PET report; for patients in ARM2, the first rater received the tau-PET report as the first exam, while the second rater received the amyloid-PET report. At T2, the two raters could revise etiological diagnosis and diagnostic confidence one last time, based on the remaining exam.

Physicians made etiological diagnoses using conventional criteria. The etiological diagnoses were categorized into two categories: (i) AD, including all the diagnoses involving AD (e.g., AD [11, 12], AD in comorbidity with cerebrovascular disease (CVD), AD with frontotemporal lobar degeneration (FTLD), AD with dementia with Lewy bodies (DLB), AD with psychiatric disorders, and AD with alcohol abuse), and (ii) non-AD, including all the remaining diagnoses without AD in comorbidity (e.g., CVD [13–15], FTLD [16], DLB [17], psychiatric disorders), suspected non-Alzheimer’s disease (SNAP) [18, 19], and normal aging.

A third rater (AM) was asked to assess the cases discordant for cognitive stage or etiological diagnosis (AD or non-AD) at baseline. Finally, the two initial raters (FA and GA) were asked to revise those cases in which their diagnoses were in disagreement with the majority, i.e., the assessment of the other two raters.

### Outcomes

The two primary study outcomes were change in the etiological diagnosis (from AD to non-AD, or from non-AD to AD) and change in diagnostic confidence across timepoints in the whole sample. Secondary outcomes included changes in diagnosis and diagnostic confidence according to the PET results. These analyses were replicated in each cognitive stage group (i.e., SCD, MCI, dementia).

### Amyloid-PET and tau-PET acquisition

Amyloid-PET scanning using [18-F] radioligands was performed at the nuclear medicine and molecular imaging division at Geneva University Hospitals with a Siemens Biograph mCT PET scanner. Amyloid-PET images were acquired using 18F-Florbetapir (*n* = 72) or 18F-Flutemetamol (*n* = 64) tracers. 18F-Florbetapir images were acquired 50 min after the intravenous administration of 200 Mbq of the radiotracer (3 × 5-min image frames). 18F-Flutemetamol images were acquired 90 min after the intravenous administration of 150 MBq of the radiotracer (4 × 5-min image frames).

Tau-PET scans were acquired using 18F-Flortaucipir (18F-AV1451), synthesized at the Center for Radiopharmaceutical Sciences ETH-PSI-USZ in Zurich, Switzerland, under license from the IP owner (Avid/Lilly). Image acquisition was performed 75 min after injection of 180 MBq of 18Fflortaucipir (6 × 5-min frames) [20].

### Amyloid-PET and tau-PET assessment

Amyloid positivity was determined in each patient by an expert in nuclear medicine (VG, > 15 years of experience in the field) using visual assessment and standard operating procedures approved by the European Medicines Agency [21, 22].

Tau distribution was determined in each patient by an expert in nuclear medicine (VG), who visually analyzed images in agreement with recently published recommendation [23], describing regions of increased 18F-Flortaucipir uptake with respect to Braak stages [24]. Further details on the visual assessment of tau-PET scans are available in Supplementary Section 2.5 in the Supplementary Material. Raters had access to PET reports. To binarize the tau-PET result in data analyses, Braak stages 0–III were considered as tau negative, and Braak stages IV–VI as tau positive, in accordance with current knowledge on the cognitive impact of tau pathology [25] and on the detectability of tau pathology with 18F-Flortaucipir [26], and consistently with a recent study [27].

### Statistical methods

Randomization into study arms was performed using a complete random assignment procedure.

Values are expressed as median (25^th^–75^th^ percentile) for continuous variables, and percentages (raw number) for the categorical variables. Sociodemographic and clinical features in ARM1 and ARM2 were compared using the Mann-Whitney test for continuous variables, and proportion test for categorical variables.

The inter-rater agreement between the blind raters for the clustered etiologic diagnoses (AD and non-AD) at the different timepoints was assessed using the unweighted Cohen’s *k* coefficient, and strength of agreement classified as *slight* (0.00–0.20), *fair* (0.20–0.40), *moderate* (0.40–0.60), *good* (0.60–0.80), and *very good* (>0.80) with 95% confidence intervals (CI). For the inter-rater agreement at baseline, we used the first assessment of the two raters (i.e., before they aligned the discordant cases with the third rater’s assessment). The non-overlap between Cohen’s *k* 95% CI was considered as statistically significant.

The McNemar chi-squared test (*χ*^2^) was used to assess changes in diagnosis (from AD to non-AD or from non-AD to AD) after PET scans. Proportion test (*χ*^2^) was used to assess the difference in diagnostic changes between pathways.

Between-pathway differences in diagnostic confidence at each timepoint and changes in diagnostic confidence along timepoints within each pathway were assessed using a linear mixed model with three repeated factors (timepoint: T1 and T2; rater: rater 1 and rater 2; pathway: AMY-TAU and TAU-AMY) and subjects as random effects; the dependent variable was the difference in diagnostic confidence between T1 and T0, and T2 and T0. We assessed the estimated marginal means of the interaction between pathway and timepoint using the Bonferroni correction to adjust *p* values of the pairwise comparisons.

All statistical analyses were performed with R, version 4.0.2 (R Foundation for statistical computing, https://www.r-project.org/).

## Results

### Participants’ features

A total of 136 patients were involved in this study. Table 1 illustrates their demographic and clinical features. Patients were on average 74 (70–78) (median, 25^th^–75^th^ percentile) years old, males and females were equally distributed. Among them, 51% (69/136) had MCI, 32% (43/136) had dementia, and 18% (24/136) had SCD. As a result of the randomization, there were no significant differences in demographic and clinical features between ARM1 and ARM2 patients (Table 1). In SCD patients with a diagnosis other than “normal aging,” the highest baseline diagnostic confidence was 85%, and dropped to 55% when focusing only on those (*n* = 3) with a baseline etiological diagnosis of AD (both values are below the threshold conventionally used to rate diagnostic confidence as “very high,” i.e., 90%). [28]

**Table 1:** Demographic and clinical features of participants

Features	Whole sample *n* = 136	ARM1 *n* = 68	ARM2 *n* = 68	ARM1 vs ARM2 *p* value
Age	74 (70–78)	73 (70–78)	74 (70–78)	0.442
Sex, male	52% (71)	51% (35)	53% (36)	1.000
Education, years	14 (11–18) [15]	14 (11–17) [8]	15 (12–18) [7]	0.551
Symptom duration, years	2 (1–4) [26]	2 (1–4) [13]	2 (1–4) [13]	0.968
MMSE	27 (24–28) [3]	27 (24–29) [1]	27 (24–28) [2]	0.392
Cognitive stage				0.185
SCD	18% (24)	23% (16)	12% (8)	
MCI	51% (69)	48% (33)	53% (36)	
Dementia	32% (43)	28% (19)	35% (24)	
Etiological diagnosis, AD	67% (91)	63% (43)	71% (48)	0.466
Amyloid status, positive	54% (73)	54% (37)	53% (36)	1.000
Tau status, positive	37% (50)	38% (26)	35% (24)	0.859
MRI				
MTA	1.0 (1.0–2.0) [33]	1.0 (1.0–2.0) [20]	1.5 (1.0–2.0) [13]	0.094
Fazekas	1.0 (1.0–2.0) [39]	1.0 (1.0–2.0) [18]	1.0 (1.0–1.5) [21]	0.175
Koedam	1.0 (1.0–1.5) [36]	1.0 (1.0–1.5) [22]	1.0 (1.0–1.5) [14]	0.916

*SCD*: subjective cognitive decline, *MCI*: mild cognitive impairment, *MTA*: medial temporal atrophy

Numbers are median (25^th^–75^th^ percentile) for continuous variables, and percentages (raw number) for the categorical variables. Square brackets [] indicate the number of missing values; reading example: MMSE score was not available for 3 patients, 1 of them in ARM1 and 2 in ARM2

Amyloid and tau status were assessed using amyloid-PET and tau-PET respectively

The baseline etiological diagnosis was inconsistent with the amyloid-PET result in 28% (38/136) of cases (i.e., 28 amyloid-negative AD, and 10 amyloid-positive non-AD patients), and with the tau-PET result in 37% (51/136) of cases (i.e., 46 tau-negative AD, and 5 tau-positive non-AD patients).

### Concordance between raters

The inter-rater agreement on the etiological diagnosis (AD or non-AD) was *fair* at baseline (71% concordance rate, unweighted *k* = 0.36, 95% CI 0.20–0.52), but increased to *good* with biomarker availability at T1 (87% concordance rate, unweighted *k* = 0.74, 95% CI 0.62–0.85) and T2 (88% concordance rate, unweighted *k* = 0.76, 95% CI 0.66–0.87).

### Change in diagnosis

In the AMY-TAU pathway, 28% (38/136; *χ*^2^ = 7.6, *p* = 0.006) of patients’ diagnoses changed after amyloid-PET when presented as the first exam (T1), and an additional 9% (12/136; *χ*^2^ = 2.1, *p* = 0.149) of cases showed a further change after tau-PET as the second exam (T2) (Table 2). In the TAU-AMY pathway, 28% (38/136; *χ*^2^ = 25.3, *p* < 0.001) of patients’ diagnoses changed after tau-PET when presented as the first exam (T1), and an additional 6% (8/136; *χ*^2^ = 3.1, *p* = 0.077) of cases showed a further change after amyloid-PET as the second exam (T2) (Table 2). These changes in diagnosis were not statistically different between the two pathways both at T1 (*χ*^2^ = 0, *p* = 1.00) and T2 (*χ*^2^ = 0.5, *p* = 0.486).

**Table 2:** Overview of the primary outcomes (changes in diagnosis and changes in diagnostic confidence) in the whole sample and in the three cognitive stage groups (SCD, MCI, dementia)

Primary outcomes		Change in diagnosis (%, *p* value)				Change in diagnostic confidence (%, *p* value)			
		Whole sample *n* = 136	SCD *n* = 24	MCI *n* = 69	Dementia *n* = 43	Whole sample *n* = 136	SCD *n* = 24	MCI *n* = 69	Dementia *n* = 43
First exam	Amyloid-PET	**28%,** ***p*** **= 0.006**	17%, *p* = 1.00	**35%,** ***p*** **= 0.008**	23%, *p* = 0.343	**+18%,** ***p*** **< 0.001**	**+23%,** ***p*** **< 0.001**	**+19%,** ***p*** **< 0.001**	**+14%,** ***p*** **< 0.001**
	Tau-PET	**28%,** ***p*** **< 0.001**	17%, *p* = 0.617	**39%,** ***p*** **< 0.001**	**16%,** ***p*** **= 0.023**	**+19%,** ***p*** **< 0.001**	**+26%,** ***p*** **< 0.001**	**+22%,** ***p*** **< 0.001**	**+10%,** ***p*** **< 0.001**
Second exam	Amyloid-PET	6%, *p* = 0.077	0%	6%, *p* = 0.134	9%, *p* = 0.617	**+4%,** ***p*** **< 0.001**	+3%, *p* = 1.00	+3%, *p* = 0.114	**+5%,** ***p*** **= 0.005**
	Tau-PET	9%, *p* = 0.149	0%	12%, *p* = 0.289	9%, *p* = 0.617	**+5%,** ***p*** **< 0.001**	**+6%,** ***p*** **= 0.007**	**+4%,** ***p*** **= 0.005**	**+ 6%,** ***p*** **= 0.001**

Bold *p* values denote statistical significance (*p* < 0.05)

**Fig. 2:**
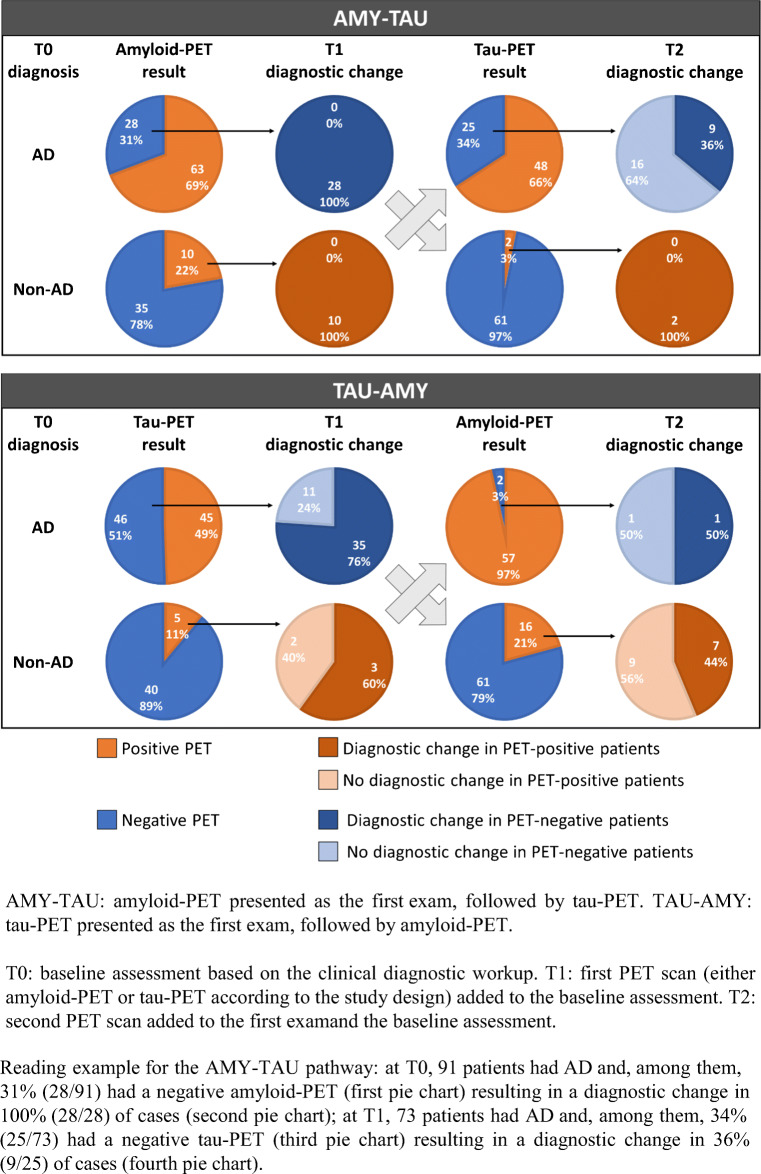
Change in diagnosis in the two diagnostic pathways (AMY-TAU and TAU-AMY).

All but one case of changes in diagnosis were observed when the PET results were inconsistent with the previous diagnosis (i.e., negative PET in AD patients, or positive PET in non-AD patients). Figure 2 illustrates how the diagnoses changed in the cases with PET results inconsistent with the previous diagnosis. In patients with a baseline diagnosis of AD and an inconsistent PET scan, 100% (28/28) changed their diagnosis to non-AD after a negative amyloid-PET, and 76% (35/46) changed to non-AD after a negative tau-PET, denoting a statistically stronger impact of a negative amyloid-PET versus a negative tau-PET (*χ*^2^ = 6.1, *p* = 0.014). In patients with a baseline diagnosis of non-AD and an inconsistent PET scan, 100% (10/10) changed their diagnosis to AD after a positive amyloid-PET, and 60% (3/5) changed to AD after a positive tau-PET (the two patients with confirmed non-AD diagnosis after positive tau-PET were both diagnosed as FTLD at baseline), a non-significantly different impact (*χ*^2^ = 1.8, *p* = 0.179) possibly due to small sample size. In patients with an etiological diagnosis of AD already supported by a first PET scan, a further change in diagnosis was observed in 36% (9/25) of patients after a negative tau-PET, and in 50% (1/2) after a negative amyloid-PET, with no difference between exams (*χ*^2^ = 0, *p* = 1.00). In patients with an etiological diagnosis of non-AD already supported by a first PET scan, a further change in diagnosis was observed in 100% (2/2) of patients after a positive tau-PET, and in 44% (7/16) after a positive amyloid-PET, with no difference between exams (*χ*^2^ = 0.56, *p* = 0.453).

Table 3 provides detailed information on the diagnostic impact of the second PET scan in both diagnostic pathways. Most of the changes in diagnosis due to amyloid-PET or tau-PET presented as the second exam were observed in patients with discordant PET results (in 6 out of 8, and 11 out of 12 cases respectively), or in patients whose baseline diagnoses were previously confirmed after the first scan (in 6 out of 8, and 9 out of 12 cases respectively).

**Table 3:** Detailed information on the diagnostic impact of the second PET scan in the two diagnostic pathways (AMY-TAU and TAU-AMY)

Diagnostic pathway	N of patients	T0 diagnosis	First PET scan	T1 diagnosis	Second PET scan	T2 diagnosis	Observations
AMY-TAU	8	AD	AMY+	AD	TAU–	Non-AD	• Discordant PET results • Same diagnosis at T0 and T1
	1	Non-AD	AMY+	AD	TAU–	Non-AD	• Discordant PET results • T1 diagnosis changed after first PET scan
	1	AD	AMY–	Non-AD	TAU+	AD	• Discordant PET results • T1 diagnosis changed after first PET scan
	1	Non-AD	AMY–	Non-AD	TAU+	AD	• Discordant PET results • Same diagnosis at T0 and T1
	1*	AD	AMY–	Non-AD	TAU–	AD	• Concordant PET results • T1 diagnosis changed after first PET scan
TAU-AMY	1	AD	TAU–	AD	AMY–	Non-AD	• Concordant PET results • Same diagnosis at T0 and T1
	4	Non-AD	TAU–	Non-AD	AMY+	AD	• Discordant PET results • Same diagnosis at T0 and T1
	2	AD	TAU–	Non-AD	AMY+	AD	• Discordant PET results • T1 diagnosis changed after first PET scan
	1	Non-AD	TAU+	Non-AD	AMY+	AD	• Concordant PET results • Same diagnosis at T0 and T1

*AMY+/−*: amyloid positive/negative, *TAU+/−*: tau positive/negative

This table includes only patients who changed diagnosis after amyloid-PET (*n* = 8) or tau-PET (*n* = 12) presented as the second exam

*The only case in which the diagnosis changed after a consistent PET result was a patient with a T1 diagnosis of MCI not due to AD based on a negative amyloid-PET whose diagnosis changed to AD at T2 after a negative tau-PET scan (Braak stage = I–III)

The only case in which the diagnosis changed after a consistent PET result was a patient with a T1 diagnosis of MCI not due to AD based on a negative amyloid-PET whose diagnosis changed to AD at T2 after a negative tau-PET scan (Braak stage = I–III) (see the “Discussion” section for further information on this case).

### Change in diagnostic confidence

**Fig. 3:**
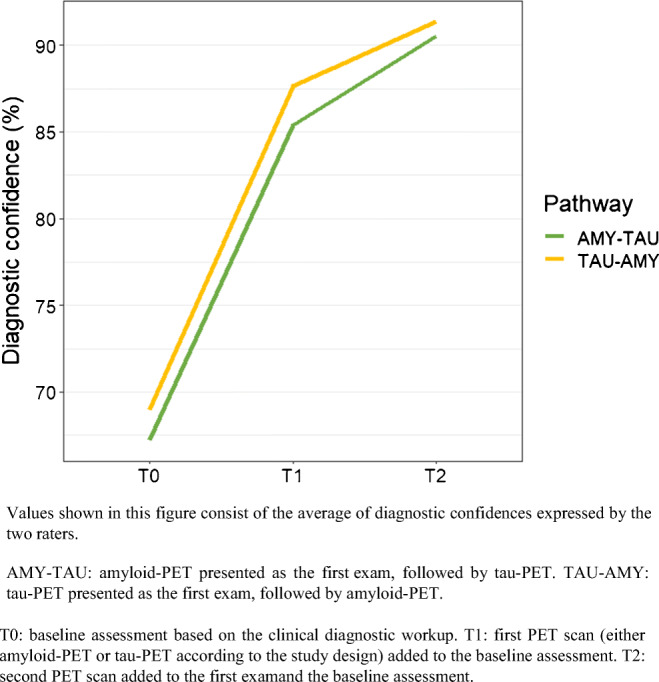
Change in diagnostic confidence in the two diagnostic pathways (AMY-TAU and TAU-AMY)

At T1, diagnostic confidence significantly increased by 18% after amyloid-PET (*p* < 0.001) and by 19% after tau-PET (*p* < 0.001) due to the first PET scan, with no difference between the two exams (*p* = 1.00). At T2, tau-PET further increased diagnostic confidence by 5% (*p* < 0.001), and amyloid-PET by 4% (*p* < 0.001), with no difference in the final diagnostic confidence between the two pathways (*p* = 1.00) (Table 2 and Fig. 3).

### Change in diagnosis and diagnostic confidence in each cognitive stage group

#### SCD

When presented as the first exam, the etiological diagnosis changed in 17% (4/24; *χ*^2^ = 0, *p* = 1.00) of SCD patients after amyloid-PET, and in 17% (4/24; *χ*^2^ = 0.25, *p* = 0.617) of SCD patients after tau-PET (Table 2), with no difference between the two exams (*χ*^2^ = 0, *p* = 1.00). The addition of a second PET scan resulted in no change in the etiological diagnosis of SCD patients both after amyloid-PET and tau-PET (Table 2). Figure S2 in the Supplementary Material illustrates how the diagnoses changed in SCD patients with PET results inconsistent with the previous etiological diagnosis.

When presented as the first exam, diagnostic confidence increased by 23% (*p* < 0.001) after amyloid-PET, and by 26% (*p* < 0.001) after tau-PET (Table 2), with no difference between the two exams (*p* = 1.00). Diagnostic confidence further increased by 3% (*p* = 1.00) after amyloid-PET and by 6% (*p* = 0.007) after tau-PET when presented as the second exams (Table 2), with no difference in the final diagnostic confidence between the two pathways (*p* = 1.00). Figure S3 in the Supplementary Material illustrates how the diagnostic confidence of SCD patients changed in the two diagnostic pathways.

#### MCI

When presented as the first exam, the etiological diagnosis changed in 35% (24/69; *χ*^2^ = 7.0, *p* = 0.008) of MCI patients after amyloid-PET, and in 39% (27/69; *χ*^2^ = 17.9, *p* < 0.001) of MCI patients after tau-PET (Table 2), with no difference between the two exams (*χ*^2^ = 0.1, *p* = 0.724). The addition of a second PET scan resulted in a further change in the etiological diagnosis in 6% (4/69; *χ*^2^ = 2.2, *p* = 0.134) of MCI patients after amyloid-PET, and 12% (8/69; *χ*^2^ = 1.1, *p* = 0.289) of MCI patients after tau-PET (Table 2), with no difference between the two exams (*χ*^2^ = 0.8, *p* = 0.365). Figure S2 in the Supplementary Material illustrates how the diagnoses changed in MCI patients with PET results inconsistent with the previous etiological diagnosis.

When presented as the first exam, diagnostic confidence increased by 19% (*p* < 0.001) after amyloid-PET, and by 22% (*p* < 0.001) after tau-PET (Table 2), with no difference between the two exams (*p* = 0.499). Diagnostic confidence further increased by 3% (*p* = 0.114) after amyloid-PET and by 4% (*p* = 0.005) after tau-PET when presented as the second exams (Table 2), with no difference in the final diagnostic confidence between the two pathways (*p* = 1.00). Figure S3 in the Supplementary Material illustrates how the diagnostic confidence of MCI patients changed in the two diagnostic pathways.

#### Dementia

When presented as the first exam, the etiological diagnosis changed in 23% (10/43; *χ*^2^ = 0.9, *p* = 0.343) of dementia patients after amyloid-PET, and in 16% (7/43; *χ*^2^ = 5.14, *p* = 0.023) of dementia patients after tau-PET (Table 2), with no difference between the two exams (*χ*^2^=, *p* = 0.588). The addition of a second PET scan resulted in a further change in the etiological diagnosis in 9% (4/43; *χ*^2^ = 0.25, *p* = 0.617) of dementia patients both after amyloid-PET and after tau-PET (Table 2), with no difference between the two exams (*χ*^2^ = 0, *p* = 1.00). Figure S2 in the Supplementary Material illustrates how the diagnoses changed in dementia patients with PET results inconsistent with the previous etiological diagnosis.

When presented as the first exam, diagnostic confidence increased by 14% (*p* < 0.001) after amyloid-PET, and by 10% (*p* < 0.001) after tau-PET (Table 2), with no difference between the two exams (*p* = 0.807). Diagnostic confidence further increased by 5% (*p* = 0.005) after amyloid-PET and by 6% (*p* = 0.001) after tau-PET when presented as the second exams (Table 2), with no difference in the final diagnostic confidence between the two pathways (*p* = 0.705). Figure S3 in the Supplementary Material illustrates how the diagnostic confidence of dementia patients changed in the two diagnostic pathways.

### Discordant amyloid-PET and tau-PET results

The amyloid-PET and tau-PET results were reciprocally discordant in 20% (27/136) of cases, with 25 patients with positive amyloid-PET and negative tau-PET (Braak stage = 0 in 14 patients, Braak stage = I–III in 11 patients), and 2 cases having patients with negative amyloid-PET and positive tau-PET (Braak stage = V in both cases). Among these discordant patients, the final (T2) diagnosis was consistent with the amyloid-PET result in 55% (15/27) of cases for the first rater and in 67% (18/27) for the second rater, and with the tau-PET result in the remaining cases.

## Discussion

In the present study, two independent raters assessed patients’ clinical reports at three different timepoints, first based only on the routine diagnostic workup (clinical and neuropsychological assessment, MRI, other exams), and then based also on the availability of the first and second PET scans in a randomized fashion. We observed that the impacts of amyloid-PET and tau-PET on diagnosis were overall similar: both PET scans induced significant and similar changes in diagnosis when presented as the first exam (28% due to amyloid-PET and 28% due to tau-PET), while the diagnostic change after the second exam was not significant for both PETs (6% due to amyloid-PET and 9% due to tau-PET). This might be partially due to the high agreement between amyloid-PET and tau-PET (80% in the present study), as compared to studies showing a moderate correlation between amyloid-PET and tau-PET in cognitively normal individuals [29]. Analyzing in detail the diagnostic impact of the second PET scan, most changes were observed in patients with discordant PET results or in patients whose baseline diagnoses were previously confirmed after the first scan, denoting that a second scan has a clinically significant impact (albeit not significant at group level) on some individuals with discordant biomarkers and even on those with a previous biomarker-based diagnosis.

All but one diagnostic change were due to PET results inconsistent with the previous etiological diagnosis. Interestingly, a negative amyloid-PET result had a stronger impact than a negative tau-PET when presented as the first exam (100% vs 76%, *p* = 0.014). The only case reclassified after a consistent PET scan was an amyloid-negative MCI patient who changed the etiological diagnosis from non-AD to AD after a tau-PET scan with Braak stage = I–III and an extensive temporal tau uptake, which one of the raters considered sufficient to have a diagnostic impact. This judgment was based on the evidence that subjects with Braak stage = III have a neuropathologic diagnosis of AD in 63% of cases [30].

 Significant and similar increases in diagnostic confidence due to amyloid-PET or tau-PET were observed when they were presented as the first or the second exams.

The above-described results were largely influenced by MCI patients, representing the largest cognitive stage group (i.e., *n* = 69, vs 24 SCD and 43 dementia). Moreover, by qualitatively analyzing the individual cognitive stage groups, we observed that (i) MCI patients were those who benefitted the most from amyloid-PET and tau-PET in terms of change in diagnosis after the first exam (in 35% and 39% of cases respectively); (ii) SCD patients showed the highest increase in diagnostic confidence after the first exam (+23% after amyloid-PET and + 26% after tau-PET), in spite of a very limited impact on diagnosis; and (iii) dementia patients showed a limited impact on diagnostic confidence after the first exam (+14% after amyloid-PET and + 10% after tau-PET). We acknowledge that these subgroup analyses should be interpreted with caution, especially for SCD and dementia patients, due to small sample sizes.

Interestingly, the availability of at least one PET is enough to significantly increase the concordance between raters from *fair* (71% concordance rate, unweighted *k* = 0.36) to *good* (87% concordance rate, unweighted *k* = 0.74).

Several studies have already assessed the diagnostic value of amyloid-PET, supporting its potential utility in clinical practice [5], while no evidence is available on the diagnostic value of tau-PET or on their combination. To the best of our knowledge, this is the first study assessing the diagnostic value of tau-PET and comparing it with that of amyloid-PET in a memory clinic population. Our findings were partially discordant with our expectation of a higher diagnostic value of tau-PET. Indeed, studies on tau-PET with 18F-Flortaucipir showed that (i) Braak stages, representing tau load and topography, better correlate with clinical symptoms than amyloid load [29, 31, 32]; (ii) its positivity indicates both advanced amyloid and tau neuropathology [23]; (iii) it proved to be able to differentiate between amyloid-positive and amyloid-negative neurodegenerative diseases with high accuracy [33]; (iv) tau deposition is closely related to other markers of neuronal injury, e.g., FDG-PET and gray matter atrophy [34–36]. On the other hand, amyloid-PET might be more sensitive than tau-PET to the early phases (amyloid is the first biomarker becoming abnormal during the AD course [37]), and, since it has been available for longer [4], it might be perceived by physicians as more reliable than tau-PET. In some cases, the raters attributed positive uptake on tau-PET to pathology other than AD-related tau, as 18F-Flortaucipir binding had been reported in frontotemporal dementia forms [38]. This, together with the higher impact on the etiological diagnosis of a negative amyloid-PET (as compared to a negative tau-PET) as shown by our data, might have rebalanced the comparison.

This study fits into the phase 3 of the biomarker roadmap for the validation for Alzheimer’s biomarkers [39] by providing pilot evidence on the *comparative* (secondary aim 2) and *combined* (secondary aim 3) diagnostic value of amyloid-PET and tau-PET.

### Limitations

The main limitation of this study is its retrospective nature. The retrospective evaluation of clinical reports does not allow, by definition, the clinical observation of the patient, depriving physicians of a key element of the diagnostic process. As a consequence, the diagnostic classifications should be considered as diagnostic hypotheses within a research setting rather than as actual clinical diagnoses. For example, the two raters involved in this study made a baseline etiological diagnosis of AD in three SCD patients based on their clinical reports. The diagnostic confidence in these AD diagnoses was never higher than 55%, corresponding to nearly the maximum uncertainty, meaning that the clinicians would not be confident in disclosing such diagnosis even if this is their diagnostic suspect. Nevertheless, the adopted study design allowed to compare and combine the diagnostic value of amyloid-PET and tau-PET in a more structured way, which is not applicable in clinical practice (where physicians usually make a diagnosis only after the conclusion of the diagnostic workup, not after each exam). Moreover, it might be interesting to follow patients over time in order to assess the impact of amyloid-PET and tau-PET on a final longitudinal diagnosis (e.g., after 2, 5, or 10 years after baseline). Unfortunately, this longitudinal information was not available at the time of the present study. A prospective longitudinal study is needed to properly address this matter.

A second limitation is the dichotomization of the tau status. Indeed, while in clinical practice the amyloid-PET result is usually dichotomized based on established cut-offs, there are no clinically validated positivity cut-offs for tau-PET, the result of which can then be described on the basis of its topography. In the present study, significant tracer deposition in brain areas corresponding to Braak stages IV–VI was classified as tau positive in accordance with current knowledge on the cognitive impact of tau pathology [25] and on the detectability of tau pathology with 18F-Flortaucipir [26], and consistently with a recent study [27]. According to a different classification method, a positive tau-PET interpretation is consistent with Braak stages V–VI [23]. We underline that, according to this classification method, only 5% (7/136) of subjects, i.e., those who were classified as Braak stage = IV, would be considered as misclassified. Thus, we can conclude that the choice of the classification method had a limited influence on our results. Nevertheless, the raters did not receive only the dichotomized tau status but also the full tau-PET report with an estimate of the Braak stage. Thus, they could consider the whole picture and make their decisions accordingly. In fact, this explains why two patients changed diagnosis from non-AD to AD after a tau-PET Braak stage = I–III, which was negative according to the study operationalization, but considered as already positive by one of the raters. Further research is needed to understand whether a dichotomous tau-PET result has a greater clinical value than a stage classification, and to validate visual assessment methods [7].

Finally, the study design prevents from drawing definitive conclusions on the diagnostic value of amyloid-PET and tau-PET, which should ideally be assessed against a gold or reference standard informing about pathology, or evidence of progression with confirmation of diagnosis at follow-up examination. Even if this study provides pilot evidence on the clinical utility of amyloid-PET and tau-PET (biomarker roadmap phase 3, secondary aims 2 and 3; and phase 4, primary aim 1), more studies on their analytical validity and clinical validity are still needed to validate their clinical use. Indeed, pathology-based studies are very rare but necessary to estimate the proportion of false-positive and false-negative cases against pathology, which will inform us about how many times clinicians will encounter a “true” AD (or non-AD) patient with inconsistent/wrong tau-PET or amyloid-PET (or both) scans.

## Conclusion

Amyloid-PET and tau-PET showed a significant and similar impact on etiological diagnosis and diagnostic confidence when presented as the first exam, although a negative amyloid-PET showed a higher impact on etiological diagnosis than a negative tau-PET. The impact of both exams was still significant in terms of improved diagnostic confidence when added as the second PET exam.

## Supplementary Information

ESM 1 (DOCX 962 kb)

## Declarations

### Disclaimer

This communication reflects the views of the authors and neither IMI nor the European Union and EFPIA are liable for any use that may be made of the information contained herein.
