# Supplementary material for: Diagnostic value of amyloid-PET and tau-PET: a head-to-head comparison
Source: Eur J Nucl Med Mol Imaging. 2021 Feb 27;48(7):2200–11. doi: 10.1007/s00259-021-05246-x (PMC8175315; doi:10.1007/s00259-021-05246-x)
Supplement: Supplementary file 1 — (DOCX 962 kb) [file 259_2021_5246_MOESM1_ESM.docx]

**Diagnostic value of amyloid-PET and tau-PET: a head-to-head comparison**

Daniele Altomare, PhD^1,2^; Camilla Caprioglio, MS^1,2^; Frédéric Assal, MD^3,4^; Gilles Allali, MD, PhD^3,5^; Aline Mendes, MD^6^; Federica Ribaldi, MS^1,7,8^; Kelly Ceyzeriat, PhD^9,10^; Marta Martins, MS^1,2^; Szymon Tomczyk, PhD^1,2^; Sara Stampacchia, PhD^9^; Alessandra Dodich, PhD^11^; Marina Boccardi, PhD^12^; Christian Chicherio, PhD^2^; Giovanni B. Frisoni, MD^1,2^; Valentina Garibotto, MD^8,13^

^1^ Laboratory of Neuroimaging of Aging (LANVIE), University of Geneva, Geneva, Switzerland.

^2^ Memory Clinic, Geneva University Hospitals, Geneva, Switzerland.

^3^ Division of Neurology, Department of Clinical Neurosciences, Geneva University Hospitals, Geneva, Switzerland.

^4^ Faculty of Medicine, University of Geneva, Geneva, Switzerland.

^5^ Department of Neurology, Division of Cognitive & Motor Aging, Albert Einstein College of Medicine, Yeshiva University, Bronx, New York, USA.

^6^ Division of Geriatrics, Department of Rehabilitation and Geriatrics, Geneva University Hospitals, Geneva, Switzerland.

^7^ Laboratory of Alzheimer’s Neuroimaging and Epidemiology (LANE), Saint John of God Clinical Research Centre, Brescia, Italy.

^8^ Department of Molecular and Translational Medicine, University of Brescia, Brescia, Italy.

^9^ Laboratory of Neuroimaging and Innovative Molecular Tracers (NIMTlab), Geneva University Neurocenter and Faculty of Medicine, University of Geneva, Geneva, Switzerland.

^10^ Division of Adult Psychiatry, Department of Psychiatry, Geneva University Hospitals, Geneva, Switzerland.

^11^ Center for Mind/Brain Sciences - CIMeC, University of Trento, Rovereto, Italy.

^12^ Late Translational Dementia Research Group, German Center for Neurodegenerative Diseases (DZNE), Rostock-Greifswald site, Rostock, Germany.

^13^ Division of Nuclear Medicine and Molecular Imaging, Geneva University Hospitals, Geneva, Switzerland.

**SUPPLEMENTARY MATERIAL**

**Supplementary Section 2.5**. Visual assessment of tau-PET scans.

**Figure S1**. Scheme depicting 4 ROIs corresponding to the neuropathological stages defined by Braak for tau deposition.

**Figure S2**. Change in diagnosis in the two diagnostic pathways (AMY-TAU and TAU-AMY) in the individual cognitive stage groups (i.e. SCD, MCI, dementia).

**Figure S3**. Change in diagnostic confidence in the two diagnostic pathways (AMY-TAU and TAU-AMY) in the individual cognitive stage groups (i.e. SCD, MCI, dementia).

**Supplementary Section 2.5**. The nuclear medicine physician was instructed through a standard visual analysis procedure on Osirix software, including PET and MRI images synchronization and display on a continuous color map (Rainbow scale) spanning the full range of intensity with the cerebellar cortex set approximately at the 30% of the color-scale. The evaluation of the images was performed mainly according to the axial view, and sagittal and coronal views could be used in support. The nuclear medicine physicians provided as output a dichotomous classification in tau negative, tau positive for an Alzheimer’s disease pattern or non-Alzheimer’s disease distribution, as well as an estimated Braak stage in case of an AD pattern, defined as follows.

Image assessment was based on a structured scheme depicting four regions of interest (ROIs) corresponding to the neuropathological stages defined by Braak for tau deposition, shown in Figure S1. The presence of tau deposition in each ROI was evaluated for each hemisphere independently, and the visually estimated Braak stage was defined considering the hemisphere showing the highest tau positivity in the ROIs evaluated. Finally, tau positivity was defined when the rater score was Braak stage ≥ IV.

**Figure S1**. Scheme depicting four regions of interest corresponding to the neuropathological stages defined by Braak for tau deposition.


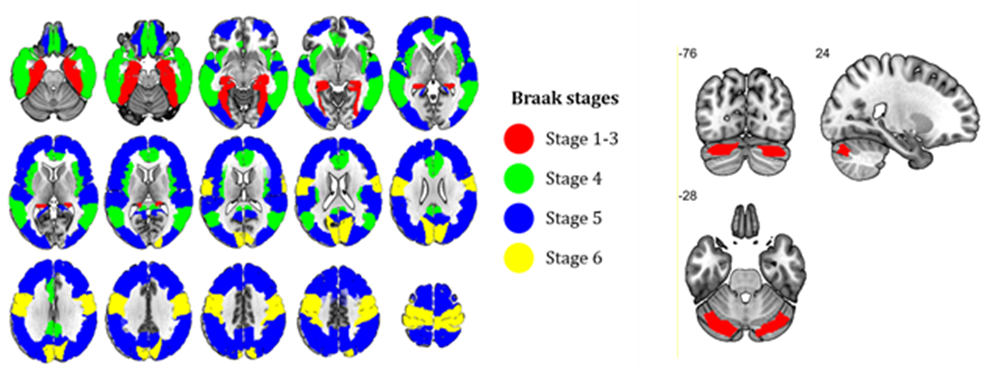


The picture represents in different colors the regions of interest (ROIs) corresponding to neuropathological Braak stages according to the tau deposition using 18F-Flortaucipir as an in vivo PET biomarker. ROIs corresponding to Braak stage I-III (red color) include medial temporal region; ROIs corresponding to Braak stage IV (green color) include anterior, inferior, mid and superior temporal regions and frontal inferior region; ROIs corresponding to Braak stage V (blue color) include parietal and frontal lobes; and ROIs corresponding to Braak stage VI (yellow color) include sensory-motor areas and visual primary cortex. Atypical presentation with medial temporal lobe sparing is considered.

**Figure S2**. Change in diagnosis in the two diagnostic pathways (AMY-TAU and TAU-AMY) in the individual cognitive stage groups (i.e. SCD, MCI, dementia).


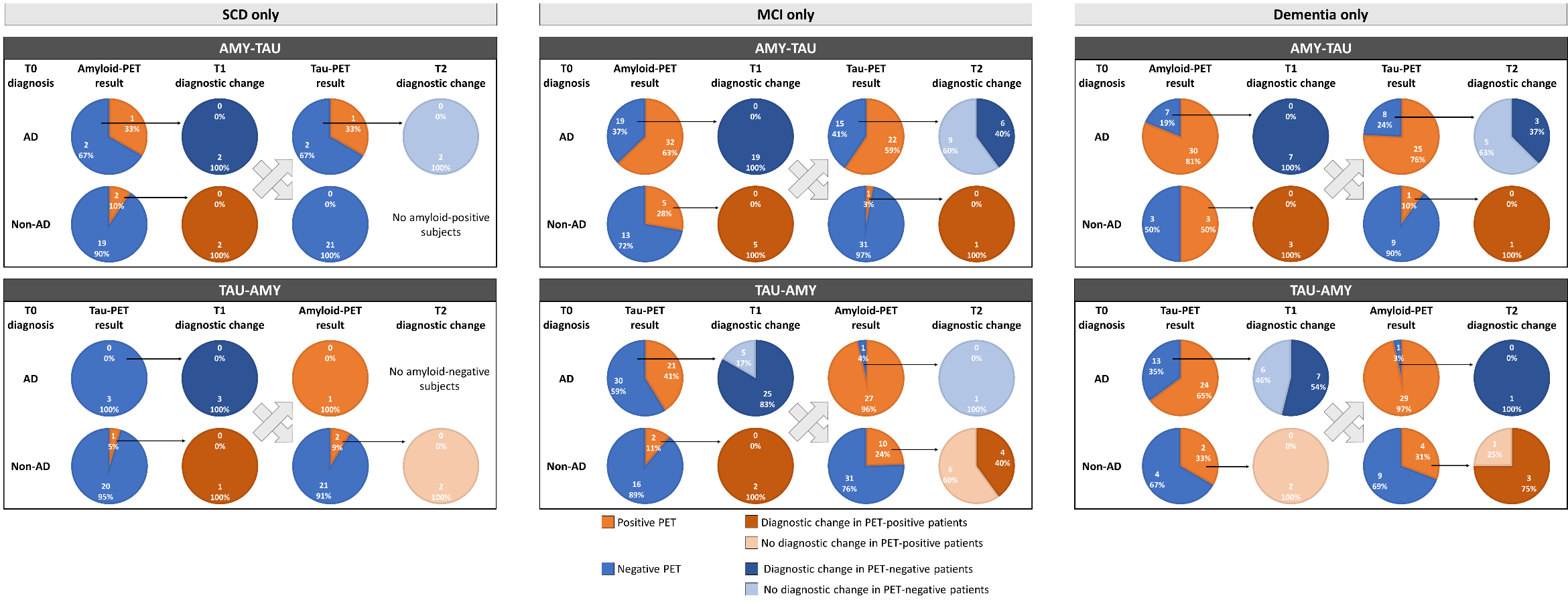


AMY-TAU: amyloid-PET presented as the first scan, followed by tau-PET. TAU-AMY: tau-PET presented as the first scan, followed by amyloid-PET.

T0: baseline assessment based on the clinical diagnostic workup. T1: first PET scan (either amyloid-PET or tau-PET according to the study design) added to the baseline assessment. T2: second PET scan added to the first scan and the baseline assessment.

Reading example for the AMY-TAU pathway in SCD: at T0, 3 SCD patients had AD and, among them, 67% (2/3) had a negative amyloid-PET (first pie chart) resulting in a diagnostic change in 100% (2/2) of cases (second pie chart); at T1, 3 SCD patients had AD and, among them, 67% (2/3) had a negative tau-PET (third pie chart) resulting in a diagnostic change in 0% (0/2) of cases (fourth pie chart).

**Figure S3**. Change in diagnostic confidence in the two diagnostic pathways (AMY-TAU and TAU-AMY) in the individual cognitive stage groups (i.e. SCD, MCI, dementia).


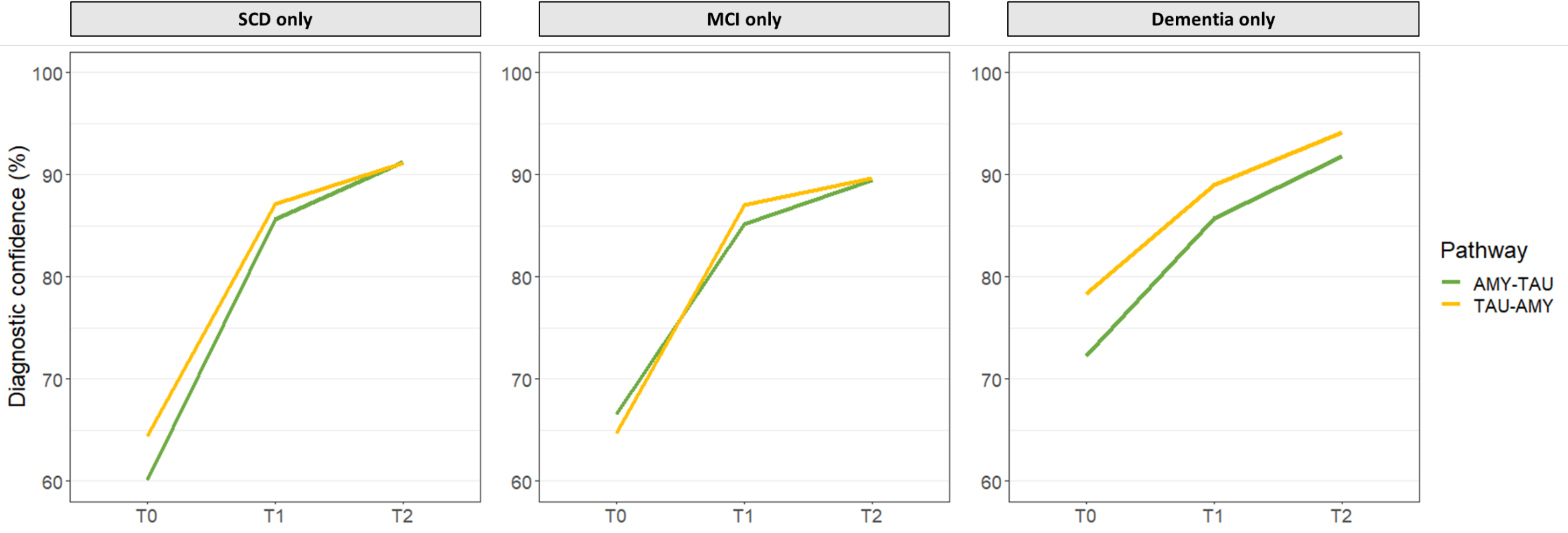


Values shown in this figure consist of the average of diagnostic confidences expressed by the two raters.

AMY-TAU: amyloid-PET presented as the first scan, followed by tau-PET. TAU-AMY: tau-PET presented as the first scan, followed by amyloid-PET.

T0: baseline assessment based on the clinical diagnostic workup. T1: first PET scan (either amyloid-PET or tau-PET according to the study design) added to the baseline assessment. T2: second PET scan added to the first scan and the baseline assessment.
